# Biological and Clinical Changes in a Pediatric Series Treated with Off-Label JAK Inhibitors

**DOI:** 10.3390/ijms21207767

**Published:** 2020-10-20

**Authors:** Alessia Pin, Alessandra Tesser, Serena Pastore, Valentina Moressa, Erica Valencic, Anna Arbo, Alessandra Maestro, Alberto Tommasini, Andrea Taddio

**Affiliations:** 1 Department of Pediatrics, Institute for Maternal and Child Health—IRCCS “Burlo Garofolo”, 34137 Trieste, Italy; alessia.pin@burlo.trieste.it (A.P.); alessandra.tesser@burlo.trieste.it (A.T.); serena.pastore@burlo.trieste.it (S.P.); valentina.moressa@burlo.trieste.it (V.M.); erica.valencic@burlo.trieste.it (E.V.); andrea.taddio@burlo.trieste.it (A.T.); 2 Department of Pharmacy and Clinical Pharmacology, Institute for Maternal and Child Health—IRCCS “Burlo Garofolo”, 34137 Trieste, Italy; anna.arbo@burlo.trieste.it (A.A.); alessandra.maestro@burlo.trieste.it (A.M.); 3 Department of Medicine, Surgery and Health Sciences, University of Trieste, 34127 Trieste, Italy

**Keywords:** Janus kinase inhibitors, off-label medications, pediatric rheumatology, interferon signature, transcriptomics, juvenile idiopathic arthritis, juvenile systemic erythematosus lupus, juvenile systemic sclerosis, monogenic interferonopathies

## Abstract

Off-label use of medications is still a common practice in pediatric rheumatology. JAK inhibitors are authorized in adults in the treatment of rheumatoid arthritis, psoriatic arthritis and ulcerative colitis. Although their use is not authorized yet in children, JAK inhibitors, based on their mechanism of action and on clinical experiences in small series, have been suggested to be useful in the treatment of pediatric interferon-mediated inflammation. Accordingly, an increased interferon score may help to identify those patients who might benefit of JAK inhibitors. We describe the clinical experience with JAK inhibitors in seven children affected with severe inflammatory conditions and we discuss the correlation between clinical features and transcriptomic data. Clinical improvements were recorded in all cases. A reduction of interferon signaling was recorded in three out of seven subjects at last follow-up, irrespectively from clinical improvements. Other signal pathways with significant differences between patients and controls included upregulation of DNA repair pathway and downregulation of extracellular collagen homeostasis. Two patients developed drug-related adverse events, which were considered serious in one case. In conclusion, JAK inhibitors may offer a valuable option for children with severe interferon-mediated inflammatory disorders reducing the interferon score as well as influencing other signal pathways that deserve future studies.

## 1. Introduction

Janus kinase (JAK) inhibitors (JAKinhibs) are small molecules with anti-inflammatory and immunosuppressive properties, due to the inhibition of Janus kinase-dependent signaling of cytokines and hormones. Three molecules received marketing authorization in humans, namely ruxolitinib, tofacitinib and baricitinib, which exhibit distinct profiles of JAK inhibition. Tofacitinib, acting on JAK1, JAK2 and JAK3 has been authorized to treat rheumatoid arthritis, psoriatic arthritis and ulcerative colitis; baricitinib, exhibiting high affinity for JAK1 and JAK2, has been authorized for rheumatoid arthritis; ruxolitinib, with a similar spectrum of baricitinib, gained market authorization to treat myelofibrosis. Even if pharmacokinetics/pharmacodynamics studies for tofacitinib and baricitinib have been performed in children, no JAKinhibs has been labelled for pediatric use so far. JAKinhibs have been used in many other conditions, such as clinical trials or off-label prescriptions, mostly in adults. The interest in this class of drugs arises from their peculiar molecular spectrum of action, targeting a distinct set of cytokines and cell functions compared with other antirheumatic drugs such as glucocorticoids and biological agents. JAKinhibs may contrast the signaling of type I and type II interferons (IFNs), which are only minimally targeted by conventional antirheumatic drugs, and interleukin 6 (IL-6). In addition, tofacitinib also significantly inhibits JAK3, reducing so far, the signaling of IL-2, IL-4, IL-7, IL-9, IL-15 and IL-21. 

Thus, even if JAKinhibs are labeled only for very few conditions, they seem to be promising candidates for precision medicine in subjects with specific inflammatory profiles, which are only partially controlled by other conventional medications. Great efforts are now being taken to obtain clinical and laboratory data suitable for patient stratification, in order to select those subjects who are more likely to benefit from a specific target drug. Transcriptomic data are considered a valuable source of information to guide the therapeutic approach in subjects with intractable disorders. Machine learning has been recently proposed as an innovative tool to classify and stratify patients with rheumatic conditions to receive precision medicines [1].

Childhood-onset diseases can provide clinical models to test the effect of the precision medicine approach; moreover, children have fewer comorbidities than adults, and early-onset disease may hide genetically impaired molecular mechanisms. For example, autoinflammatory disorders have been considered as the exemplar to unveil the great potential of anti-IL-1 therapies, and, in a similar manner, interferonopathies represent the most exemplary model to disclose the anti-inflammatory potential of JAKinhibs. 

These drugs have been anecdotally used with benefit in rare and severe disorders, paving the way for the development of phase II or III clinical trials. Albeit with a limited number of patients, pediatric case series can thus provide an ideal setting to study the effect of medications acting on relevant pathogenic mechanisms, especially if a detailed biological documentation is provided together with clinical data [2].

We describe the off-label use of JAKinhibs in early-onset pediatric inflammatory disorders and describe the outcome of patients on both clinical symptoms and transcriptomic profile changes in peripheral blood cells.

## 2. Results

### 2.1. Patients

We enrolled all the subjects who underwent treatment with a JAKinhib for a refractory inflammatory disorder at the Institute for Maternal and Child health IRCCS Burlo Garofolo. Overall, seven subjects were included in the study (six females; mean age 12.5 years, range 7–19 years). The mean age at disease onset and at the start of treatment were respectively 5 years (range 2–11 years) and 11 (range 6–17 years). All the patients had tried several medications before JAKinhibs (mean 5.1, range 3–9 medications), including conventional antirheumatic drugs and biologics (see Table 1). The mean duration of therapy with JAKinhibs at follow-up was 21.45 months (range 1–47.2 months).

### 2.2. Case Series

Patient #1 is a boy, who presented with a neonatal TORCH-like syndrome characterized by liver cirrhosis and multilinear cytopenia. In the following years, the liver disease showed spontaneous improvement, while novel symptoms occurred, such as unexplained fever spikes and chilblains. From the age of five years, he presented polyarticular arthritis, with contractures and deformities at his hands, reminiscent of Jaccoud’s arthritis, which was progressive despite several therapeutic attempts with conventional and off-label antirheumatic drugs. He also complained of severe headaches and lipodystrophy, and refractory feet ulcers hindering walking. On lab examinations, he showed only mild increases of erythrocyte sedimentation rate (ESR) and C-reactive protein (CRP), anemia and leukopenia, and inconstant antinuclear antibody positivity. At the age of 15, he was dependent on glucocorticoids, and the use of a wheelchair was proposed because of severe foot pain when standing or walking. His height was much shorter than expected, but his bone age was also delayed. Given the diagnosis of monogenic lupus-like interferonopathy and considering a strikingly elevated IFN score, we started off-label use of JAKinhibs, in combination with two antimalarials (hydroxychloroquine and mepacrine), allowing prompt resolution of headaches, significant improvement of arthritis, sparing of glucocorticoids and growth-hormone induced catch-up. Acute phase reactants became persistently normal, but a worsening of lymphopenia was observed, without the development of severe infections. Laboratory findings of a baseline point (before JAKinhibs), during JAKinhibs therapy (after JAKinhibs) and at the last follow-up (last FUP) are reported in Appendix A, Table A1.

Patient #2 is a girl presenting in her first years with chilblains, nodules at her feet, Gottron-like papules on the hands, skin rashes, livedo reticularis, arthritis, and panniculitis resulting in areas of lipodystrophy. She also presented fatigue and growth delay. She was firstly diagnosed with undifferentiated connective disease with aspects of chilblain lupus. Polyarthritis and lipodystrophy were her major complaints, which were refractory to several anti-inflammatory treatments, including antimalarials and biologics. At the age of 17, she also developed alopecia. On laboratory examinations, the girl showed persistently raised ESR associated with striking increase of the IFN score. A skin biopsy showed neutrophilic dermatosis. Based on these findings a diagnosis of CANDLE syndrome (chronic atypical neutrophilic dermatosis with lipodystrophy and elevated temperature) was made, even if no mutation was found in proteasome-related genes by exome sequencing. Treatment with tofacitinib 5 mg twice daily was started at the age of 17 years, based on favorable results reported in a few cases with CANDLE syndrome (26137574, trial NCT01724580). The treatment was associated with complete disappearance of panniculitis and resolution of alopecia, together with significant improvement in articular function. The only adverse event recorded was a transient increase of gamma glutamyl-transferase, and a dyslipidemia that was managed with low dose atorvastatin. Laboratory findings before JAKinhibs, after JAKinhibs and at the last FUP are reported in Appendix A, Table A2.

Patient #3 is a four-year-old girl who received the diagnosis of COPA syndrome after her older sister, who was affected by polyarticular arthritis and died from the same syndrome at the age of 23 years, due to hemorrhagic alveolitis and heart failure. The girl developed antinuclear antibodies positive polyarticular arthritis at the age of three years, and dry cough associated with cystic alveolar disease. Her laboratory examinations always showed increased ESR and CRP and, despite treatments, her arthritis was evolving with significant limitations. The IFN score was very high. Due to refractories to a combination therapy with methotrexate, mycophenolate acid and glucocorticoids, we stopped methotrexate and started a treatment with baricitinib. After six months of treatment, we observed a good control of articular symptoms, albeit without significant changes in acute phase reactants. After 15 months, there was no sign of articular inflammation, and acute phase reactants were in normal ranges. Laboratory findings before JAKinhibs, after JAKinhibs and at the last FUP are reported in Appendix A, Table A3.

Patient #4 is a nine-year-old girl with polyarticular juvenile idiopathic arthritis (JIA). In her first years she presented progressive joint contractures and limitations, raising the suspicion of congenital arthrogryposis. However, at the age of eight years, she received the diagnosis of polyarticular JIA. Given the severe course of the disease despite various attempts with biologic drugs, and considering a borderline IFN score, we started off-label treatment with 5 mg twice daily tofacitinib. A clear improvement of joint movements and gait was evident just two months after the start of the treatment, even if no effect was recorded on acute phase reactants. At the last follow-up no joint displayed active arthritis. Laboratory findings before JAKinhibs, after JAKinhibs and at the last FUP are reported in Appendix A, Table A4. 

Patient #5 is a girl who presented at the age of two years with recurrent painful panniculitis nodules and fevers. Episodes initially responded to short courses of low-dose corticosteroids, but symptoms worsened over the following years requiring continuous steroidal therapy. Skin biopsy was consistent with Weber–Christian panniculitis. Laboratory examinations showed persistent increase of ESR and to a lower extent of CRP. The IFN score was very high. Several attempts to spare steroids included immunosuppressants and biologic agents, with only partial benefit. A good control of her symptoms could be achieved only with baricitinib, allowing the suspension of glucocorticoids and the halving of cyclosporin A dosage. Laboratory findings before JAKinhibs, after JAKinhibs and at the last FUP are reported in Appendix A, Table A5.

Patient #6 is a girl who was referred to our Institute at the age of nine years because of fatigue, headaches, low degree fever, oral mucositis, arthralgias and butterfly rash. Laboratory investigations revealed increased aminotransferases, high ESR and positive antinuclear antibodies and SSA (anti-Sjögren’s syndrome type A) antibodies, leading to the diagnosis of systemic lupus erythematosus (SLE). Whilst most symptoms well responded to a standard combination therapy for lupus, headaches, fatigue and dysphoria remained her major complaints, with poor response to medications. Eye examination, brain MRI and brain positron emission computed tomography were normal. Conversely, spinal puncture held positive SSA antibodies in cerebrospinal fluid, and peripheral blood IFN score was very high. Given the potential role of IFN in neuropsychiatric symptoms [3] we proposed a treatment with baricitinib and tapered glucocorticoids. The treatment was associated with significant improvement in the girl’s mood and normalization of ESR. Laboratory findings before JAKinhibs, after JAKinhibs and at the last FUP are reported in Appendix A, Table A6.

Patient #7 is a girl with a history of Raynaud’s disease and antinuclear antibodies in the last three years, followed by slow and progressive development of skin hardening and joint stiffness, leading to the diagnosis of juvenile systemic sclerosis at the age of 13 years. Even if no clear systemic involvement was evident, a spirometry showed a reduced airflow, supporting an initial restrictive lung involvement. Laboratory investigations, with high-titer SCL70 antibodies (antitopoisomerase I), raised acute phase reactants and increased IFN score, were consistent with an early phase of diffuse cutaneous systemic sclerosis. Treatment was started with low-dose glucocorticoids and mycophenolic acid. A trial with rituximab was stopped due to an early drug reaction after the infusion of a few drops of the medication. Given the evidence of IFN mediated inflammation and considering anecdotal experiences from the literature, we proposed a treatment with tofacitinib. After one month of treatment, a significant improvement in the range of motion of her wrists and elbow was noticed, together with normalization of acute phase reactants. A slight further improvement occurred over the following three months of treatment, with complete recovery of elbow arthritis and with a reduction of the Modified Rodnan Score from 39 at baseline to 28 and a reduction of the Juvenile Systemic Sclerosis Severity Score from 11 to 7 [4]. Laboratory findings before JAKinhibs, after JAKinhibs and at the last FUP are reported in Appendix A, Table A7.

### 2.3. JAKinhibs Effect Reflected by Laboratory and Clinical Findings

**Figure 1:**
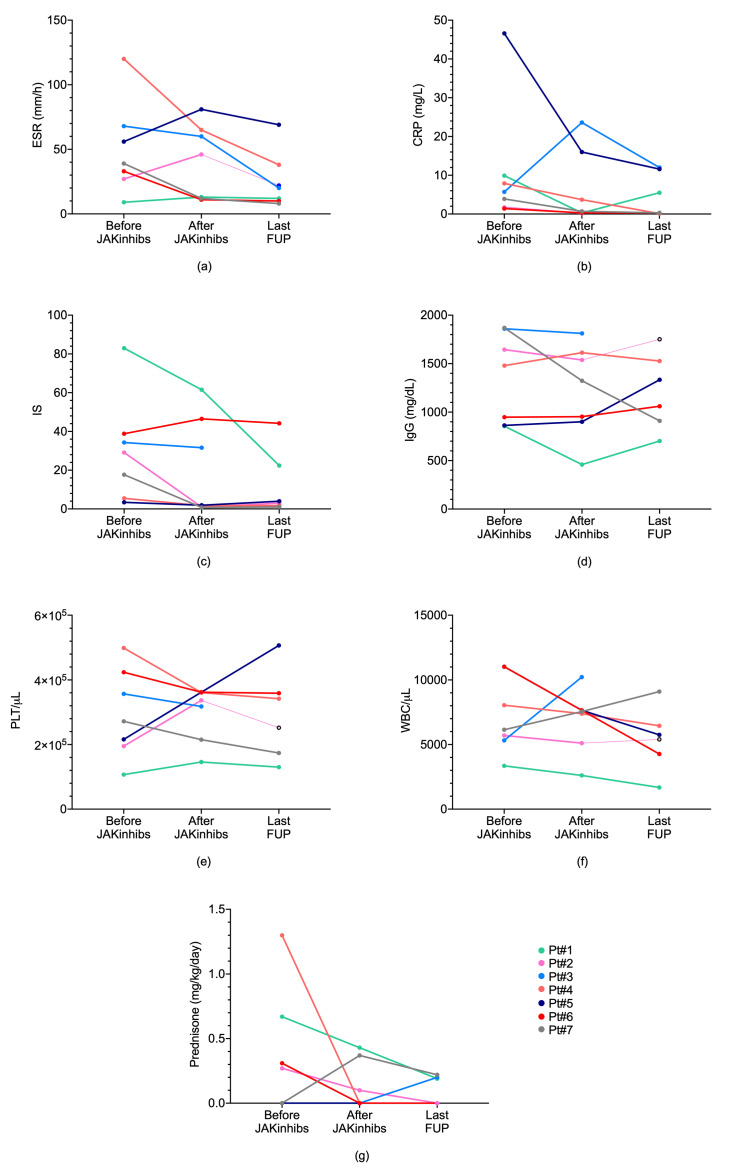
Laboratory findings and corticosteroid dosage before and after treatment with Janus kinase (JAK) inhibitors (JAKinhibs) in the seven subjects (pt 1–7). (**a**) ESR (**b**) CRP (**c**) IS (**d**) IgG (**e**) PLT (**f**) WBC (**g**) corticosteroid dosage. ESR, erythrocyte sedimentation rate; CRP, C-reactive protein; IS, interferon score; IgG, immunoglobulins; PLT, platelets; WBC, white blood count; pt, patient.

ESR (Figure 1a), CRP (Figure 1b), IFN score (IS) (Figure 1c), immunoglobulins (IgG) (Figure 1d), platelets (PLT) (Figure 1e), and white blood cells (WBC) (Figure 1f) were measured in the seven subjects included in the study: before JAKinhibs administration (baseline, RNAseq sample), and two samples during JAKinhibs therapy; an intermediate point of which RNAseq is available (after JAKinhibs, RNAseq sample) and at the last follow-up (last FUP). Overall, after JAKinhibs, inflammation indexes tended to improve in most patients. Considering the improvement of the acute phase reactants as the normalization of either ESR or CRP without worsening the other one, an overall amelioration was seen in four patients. Notably, these results were achieved in most cases despite a reduction of glucocorticoids dosage. In one case, who was enrolled at the time of diagnosis of juvenile systemic sclerosis, glucocorticoids were started as part of the standard immunosuppressive therapy, after the baseline sampling for RNAseq (Figure 1g).

### 2.4. JAKinhibs Effect Reflected on Gene Expression Patterns

#### 2.4.1. Transcriptomic Profile in Patients Compared with Control-Group Subjects: Cluster Analysis

An unsupervised nonhierarchical cluster analysis algorithm was performed comparing patients’ and healthy controls’ gene expression data, considering the expression of 500 most variable protein-coding genes across all samples. The analysis allowed defining overall transcriptomic profiles reflecting either the healthy or the diseased condition (healthy-like cluster or H-cluster and diseased-like cluster or D-cluster). Each patient was then assigned to the cluster with closer transcriptomic similarity. 

The analysis was performed on data from samples obtained both before and after JAKinhibs treatment. The most common genes involved in IFN-related pathways were excluded from the analysis [5,6] to focus on additional features shared by the seven rheumatologic patients. 

**Figure 2:**
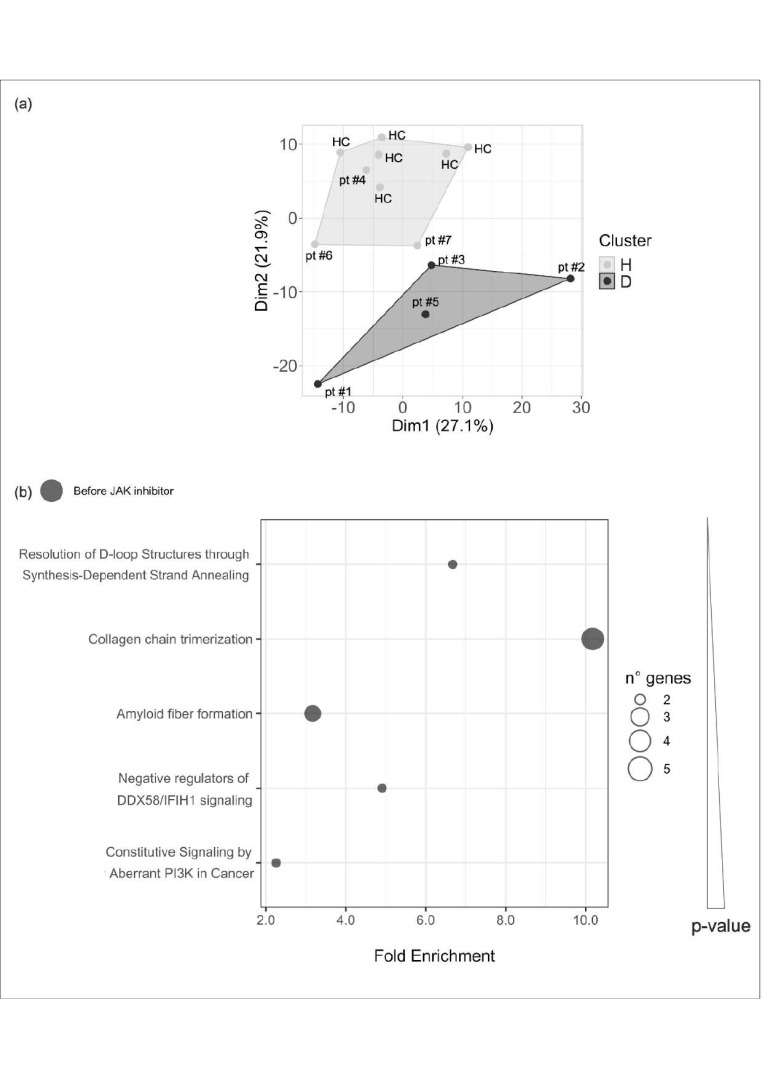
Cluster analysis and pathway enrichment. (**a**) Cluster analysis results (K-means clustering) considering 500 most variable protein-coding genes across all samples that divide subjects into subgroups by similarities. Each dot represents a subject; Dim1 and Dim2 show the higher differences between the main clusters; H-Cluster contains all the healthy control-group subjects (HC) and some patients (pt) affected with multifactorial disorders. D-Cluster contains mainly subjects with proven or suspected rare monogenic disorders. (**b**) Pathway analysis was run on differential expressed genes resulting from the comparison between the seven patients and the control-group subjects: each dot represents a pathway and the size is directly proportional to the number of genes. *X*-axis reports pathway fold enrichment. All the selected pathways showed a lowest adjusted-*p*-value of the given term over all iterations ≤0.01. Pathway are listed by increasing *p*-values.

Clustering in Figure 2a displays patients’ distribution into diseased-like (D) and healthy-like (H) subgroups before JAKinhibs treatment: H-cluster (including all the true healthy controls and patients #4, #6, and #7) and D-cluster (patients #1, #2, #3, and #5). 

D-cluster includes patients with higher systemic involvement or with rare monogenic disorders. Patients #6 and # 7 were assigned to H-cluster by the unsupervised approach. The interpretation of this result might be attributable to the presence of an intermediate gene expression features between the two clusters or to the algorithm that may be not able to discriminate some differences probably due to a limited number of samples.

**Figure A1:**
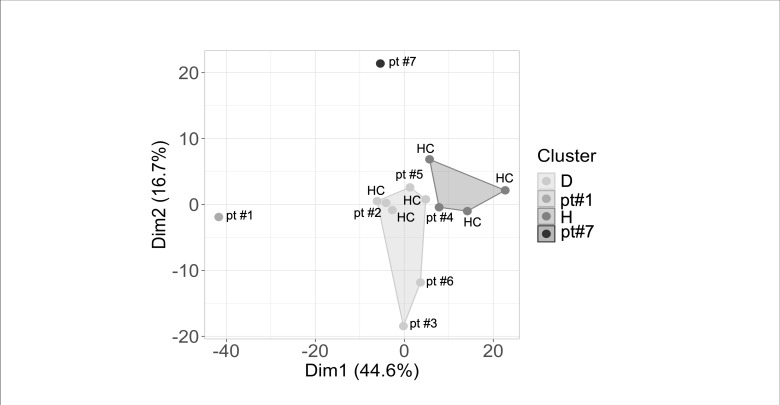
Cluster analysis of patients (pt) after JAKinhibs treatment and healthy control-group subjects (HC). Cluster analysis results (K-means clustering) considering 500 most variable protein coding genes across all samples that divide subjects into subgroups by similarities. Each dot represents a subject; Dim1 and Dim2 show the higher differences between the main clusters; D-Cluster contains three HC and patients #2, #3, #5, #6. H-Cluster 3: three HC and patient #4. Patient #1 and patient #7 are outliers.

After JAKinhib, the differences between clusters were narrowed, so much that the two main clusters, diseased-like or healthy-like, slightly changed. However, patients #1, #3, #6, and #7 still remained aside from controls (Appendix B, Figure A1).

#### 2.4.2. Transcriptomic Profile in Patients Compared with Control-Group Subjects: Pathway Analysis

Pathway analysis was run on differentially expressed genes (DEGs) resulting from the comparison between the seven patients and the control group. Figure 2b reported the most significant pathways enriched in DEGs in pretreatment patients’ samples compared with healthy individuals. For example, DNA repair pathway was upregulated in patients, with an increased expression of BRCA2 and RMI2; extracellular collagen homeostasis signaling was altered in patients at baseline; the impairments of amyloid, DDX58 (RIG-1)/IFIH and PI3K pathways are probably directly or indirectly related to inflammatory conditions. None of these pathways remained significantly altered after therapy.

#### 2.4.3. Enriched Pathways before and after JAKinhibs Therapy in Each Patient

Pathway enriched analysis focusing on the shared signaling before and after JAKinhibs was performed analyzing each patient separately. 

Pathways enriched before treatment are expected to reflect either disease-specific mechanisms or the effect of concomitant medications. Conversely, pathways altered after JAKinhibs are thought to reflect the effect of the treatment.

**Figure 3:**
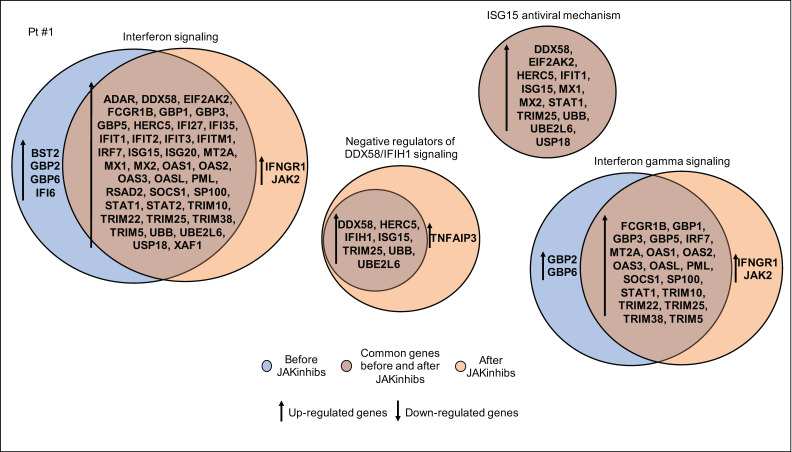
Pathway enrichment analysis before and after JAKinhibs treatment. Pt, patient.

In patient #1, IFN-stimulated genes remained significantly hyperrepressed during the treatment. After therapy, the IFN gamma receptor IFNGR1 and the kinase JAK2 appeared significantly hyperrepressed compared with controls, suggesting the establishment of an adaptive feedback in response to reduced JAK signaling. In addition, there was an increased expression of TNFAIP3 that could be speculated to play a role in inhibiting the NF-kB-mediated inflammatory cascade (Figure 3).

**Figure 4:**
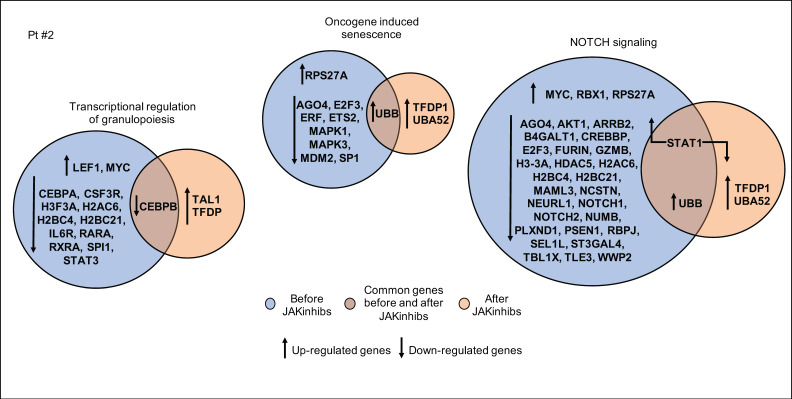
Pathway enrichment analysis before and after JAKinhibs treatment. Pt, patient.

In patient #2, genes involved in the regulation of granulopoiesis remained significantly represented during the treatment, although to a lesser extent after therapy. The upregulation of STAT1 before JAKinhibs highlights the IFN activation, which was indeed reduced after the treatment. In addition to a wider modulation of the inflammatory response, altered expression of senescence pathway-related genes before therapy may suggest a cell proliferation surveillance (Figure 4).

**Figure 5:**
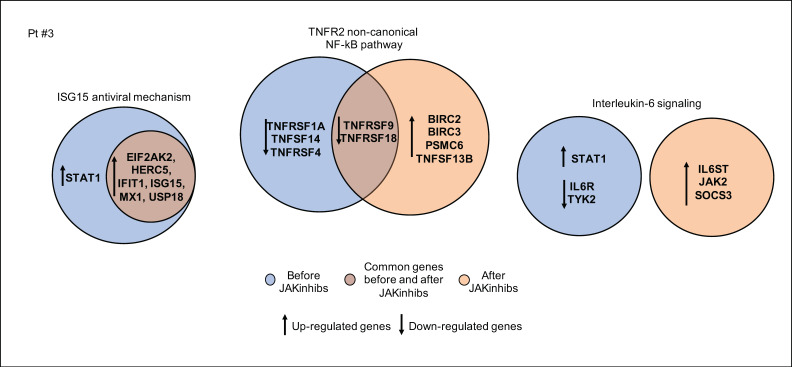
Pathway enrichment analysis before and after JAKinhibs treatment. Pt, patient.

In patient #3, the noncanonical NF-kB pathway is consistently engaged as well as the IL-6 signaling. The downregulation of IL6R before JAKinhibs, may reflect previous pharmacological treatments (Figure 5). 

Patient #4 and #5 didn’t show common altered pathways before and after therapy.

**Figure 6:**
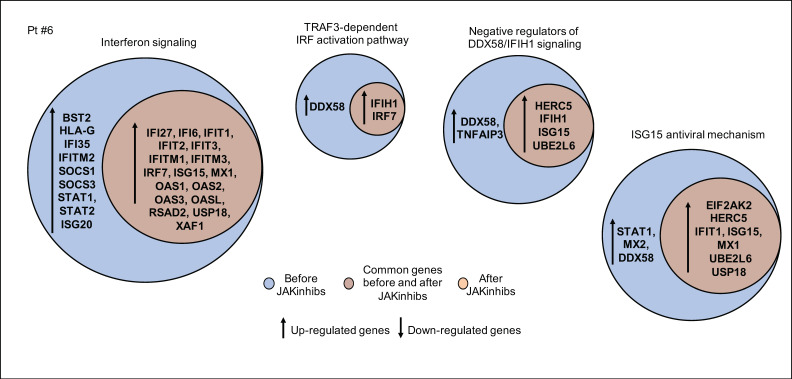
Pathway enrichment analysis before and after JAKinhibs treatment. Pt, patient.

In patient #6, despite JAKinhibs therapy, IFN signaling components remained overexpressed during the disease course (Figure 6).

**Figure 7:**
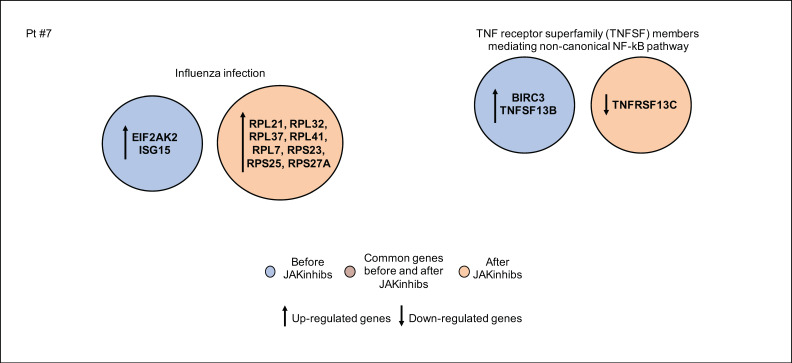
Pathway enrichment analysis before and after JAKinhibs treatment. Pt, patient.

In patient #7, a modulation of NF-kB-mediated inflammatory cascade in response to JAKinhibs was observed. The antiviral response was hyperactivated, showing an increased expression of genes involved in protein synthesis (Figure 7).

Overall, in three out of seven patients’ had overexpressed IFN-related pathway, consistently with the high IFN score detected both at the baseline and after JAKinhibs treatment, no other commonly represented signaling was pointed out between all the seven patients, according to pathfindR gene ontology analysis. The list of genes of each pathway is reported in Supplementary Table S1. Genes shared by two or more subjects are highlighted.

#### 2.4.4. Pathways Enriched of Differentially Expressed Genes Either before or after JAKinhibs in Each Patient

**Figure 8:**
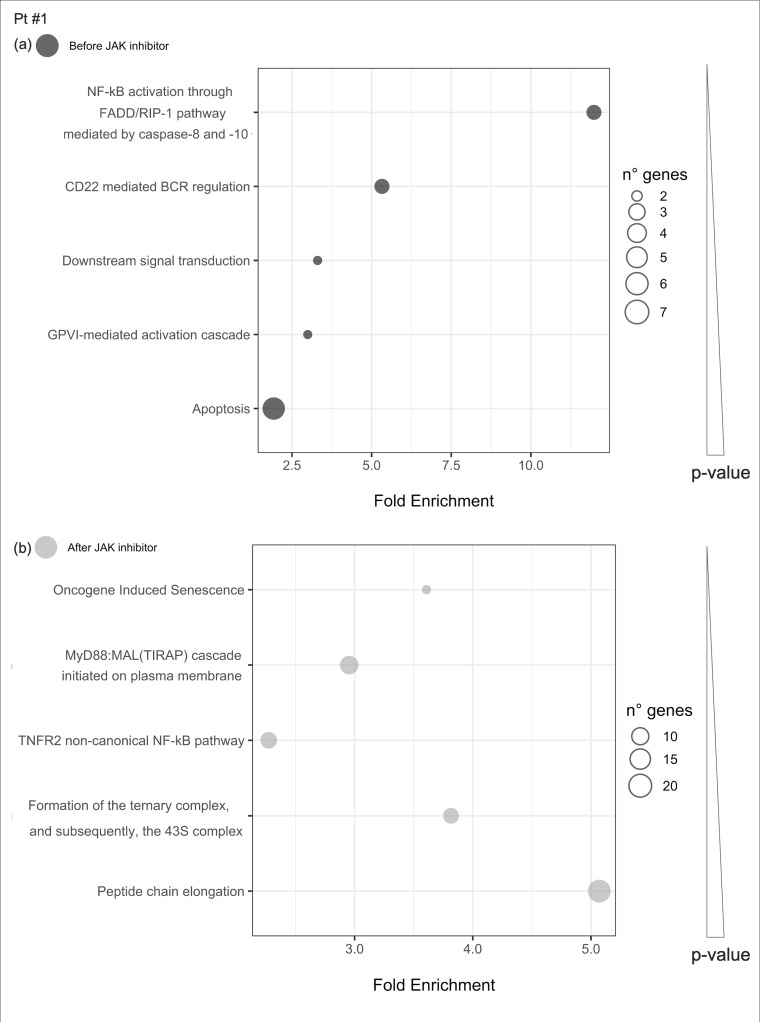
Pathway enrichment analysis was run on differential expressed genes resulting from the comparison between patient #1 before JAKinhibs and the control-group subjects and between patient #1 after JAKinhibs and the controls. Pathways altered uniquely before (**a**) or uniquely after (**b**) JAKinhibs were chosen. Each dot represents a pathway, and the size is directly proportional to the number of genes. On the *X*-axis the pathway fold enrichment is displayed. All the selected pathways showed a lowest adjusted *p*-value of the given term over all iterations ≤0.01, and the five most enriched pathways were elected, where applicable. Pathways are ordered by increasing *p*-values. Pathways altered before JAKinhibs are colored in dark grey and those altered after JAKinhibs in light grey.

**Figure 9:**
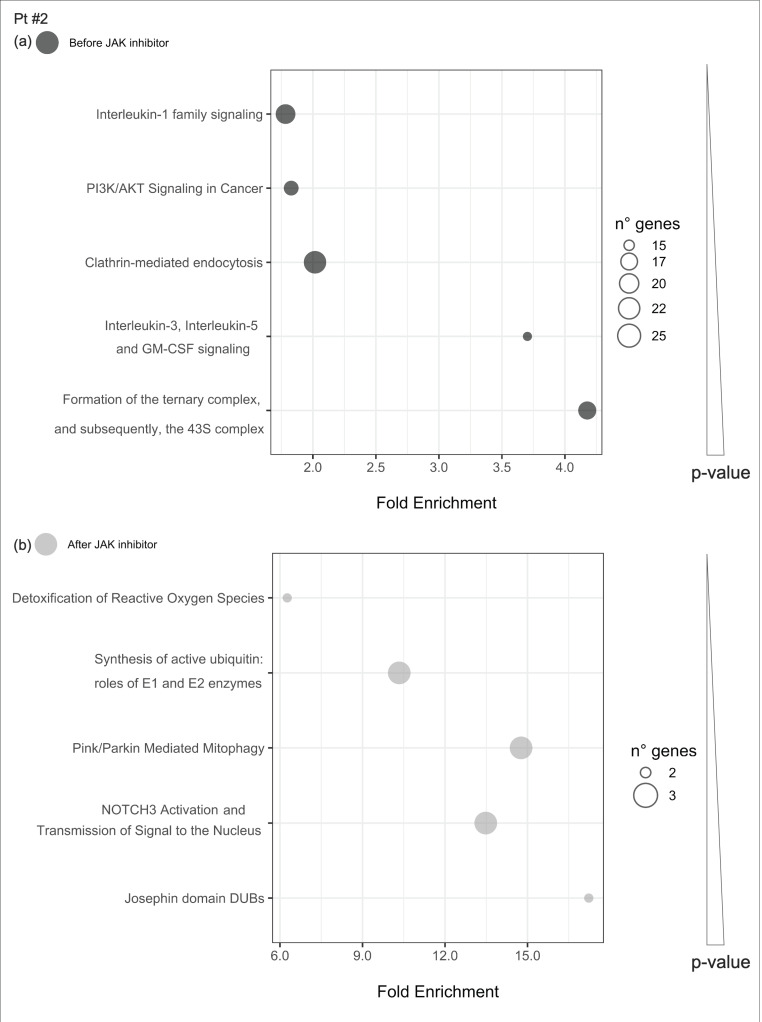
Pathway enrichment analysis was run on differential expressed genes resulting from the comparison between patient #2 before JAKinhibs and the control-group subjects and between patient #2 after JAKinhibs and the controls. Pathways altered uniquely before (**a**) or uniquely after (**b**) JAKinhibs were chosen. Each dot represents a pathway, and the size is directly proportional to the number of genes. On the *X*-axis the pathway fold enrichment is displayed. All the selected pathways showed a lowest adjusted *p*-value of the given term over all iterations ≤0.01, and the five most enriched pathways were elected, where applicable. Pathways are ordered by increasing *p*-values. Pathways altered before JAKinhibs are colored in dark grey and those altered after JAKinhibs in light grey.

**Figure 10:**
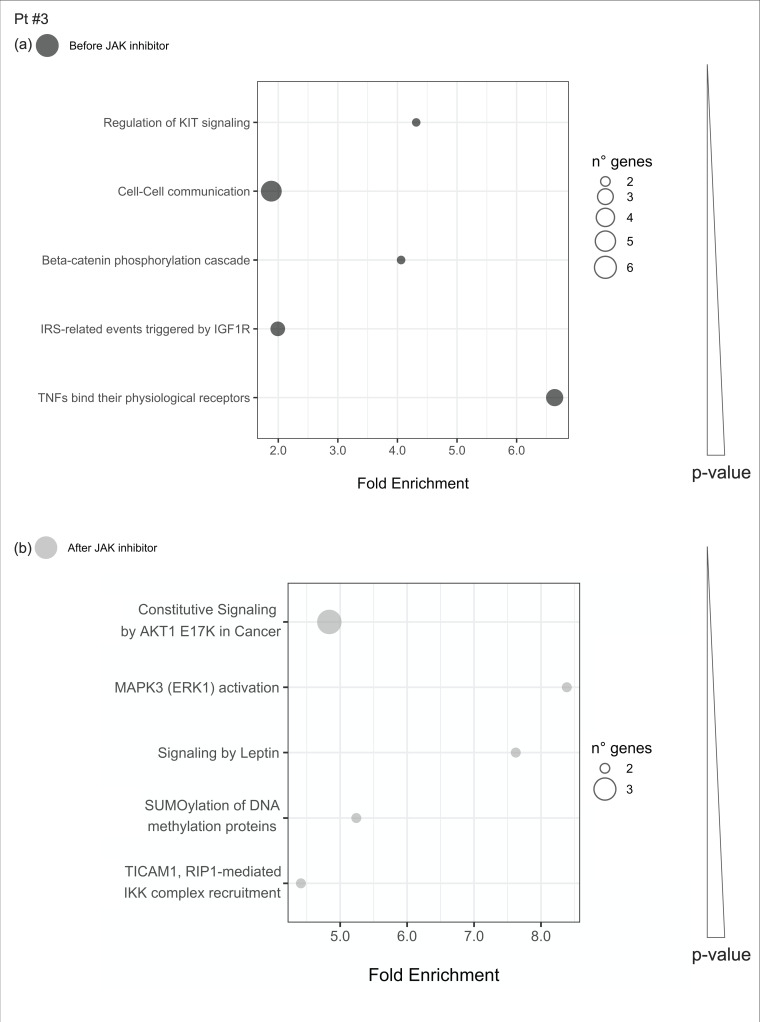
Pathway enrichment analysis was run on differential expressed genes resulting from the comparison between patient #3 before JAKinhibs and the control-group subjects and between patient #3 after JAKinhibs and the controls. Pathways altered uniquely before (**a**) or uniquely after (**b**) JAKinhibs were chosen. Each dot represents a pathway, and the size is directly proportional to the number of genes. On the *X*-axis is displayed the pathway fold enrichment. All the selected pathways showed a lowest adjusted *p*-value of the given term over all iterations ≤0.01, and the five most enriched pathways were elected, where applicable. Pathways are ordered by increasing *p*-values. Pathways altered before JAKinhibs are colored in dark grey and those altered after JAKinhibs in light grey.

**Figure 11:**
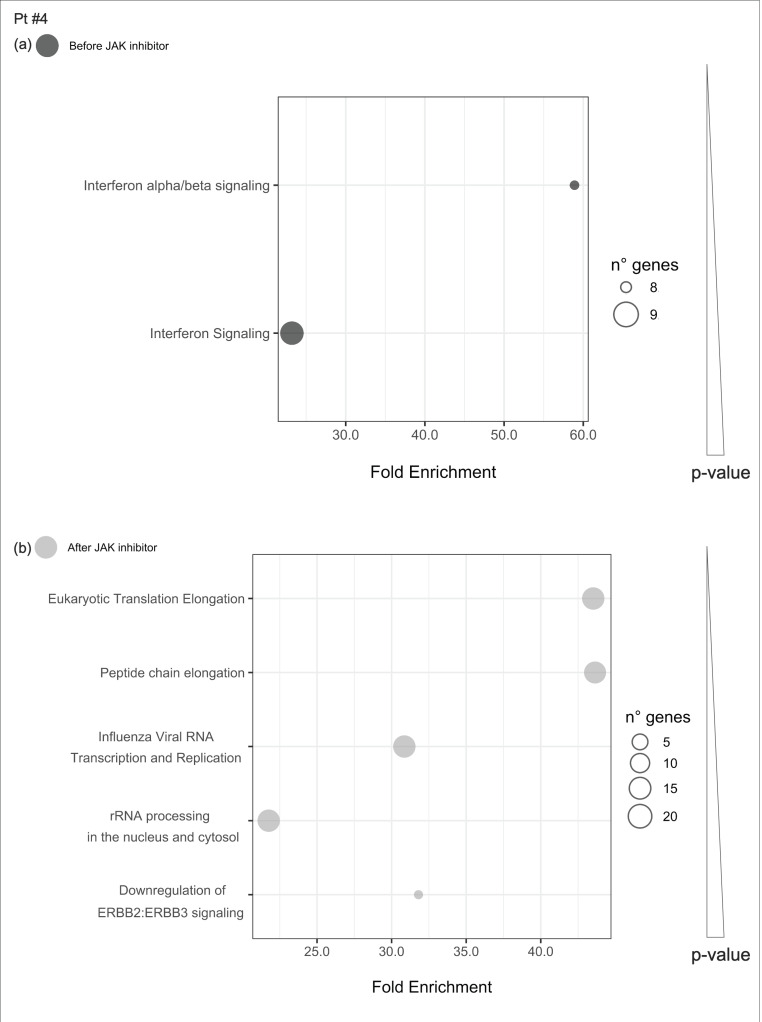
Pathway enrichment analysis was run on differential expressed genes resulting from the comparison between patient #4 before JAKinhibs and the control-group subjects and between patient #4 after JAKinhibs and the controls. Pathways altered uniquely before (**a**) or uniquely after (**b**) JAKinhibs were chosen. Each dot represents a pathway, and the size is directly proportional to the number of genes. On the *X*-axis the pathway fold enrichment is displayed. All the selected pathways showed a lowest adjusted *p*-value of the given term over all iterations ≤0.01, and the five most enriched pathways were elected, where applicable. Pathways are ordered by increasing *p*-values. Pathways altered before JAKinhibs are colored in dark grey and those altered after JAKinhibs in light grey.

**Figure 12:**
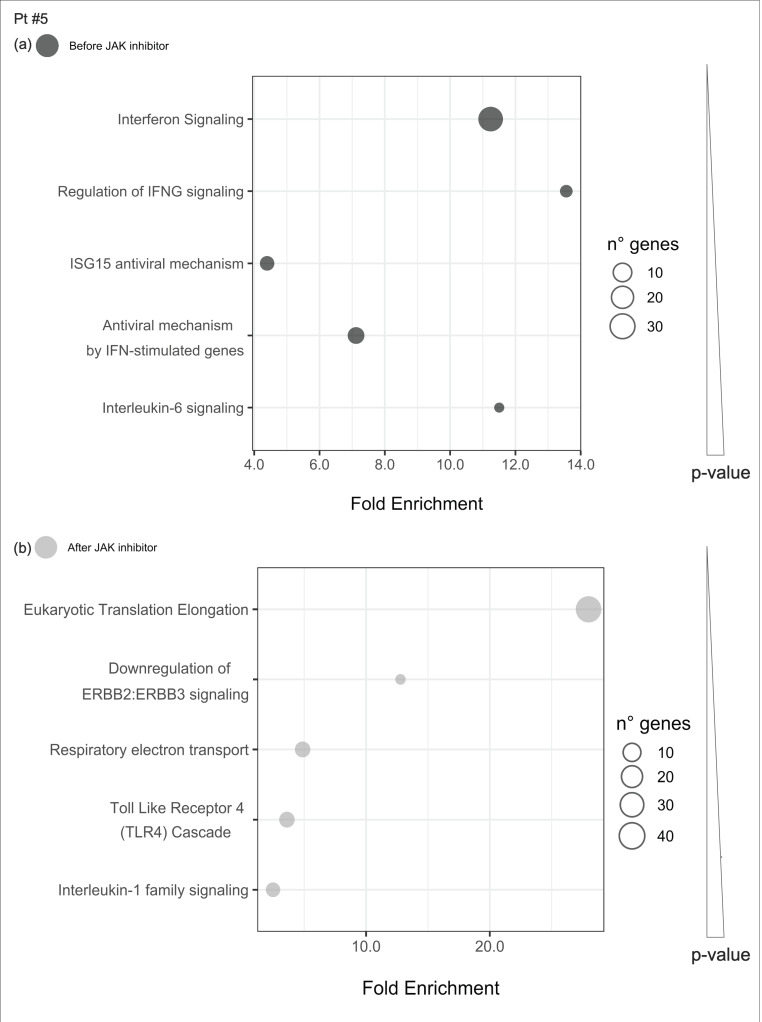
Pathway enrichment analysis was run on differential expressed genes resulting from the comparison between patient #5 before JAKinhibs and the control-group subjects, and between patient #5 after JAKinhibs and the controls. Pathways altered uniquely before (**a**) or uniquely after (**b**) JAKinhibs were chosen. Each dot represents a pathway, and the size is directly proportional to the number of genes. On the *X*-axis the pathway fold enrichment is displayed. All the selected pathways showed a lowest adjusted *p*-value of the given term over all iterations ≤0.01, and the five most enriched pathways were elected, where applicable. Pathways are ordered by increasing *p*-values. Pathways altered before JAKinhibs are colored in dark grey and those altered after JAKinhibs in light grey.

**Figure 13:**
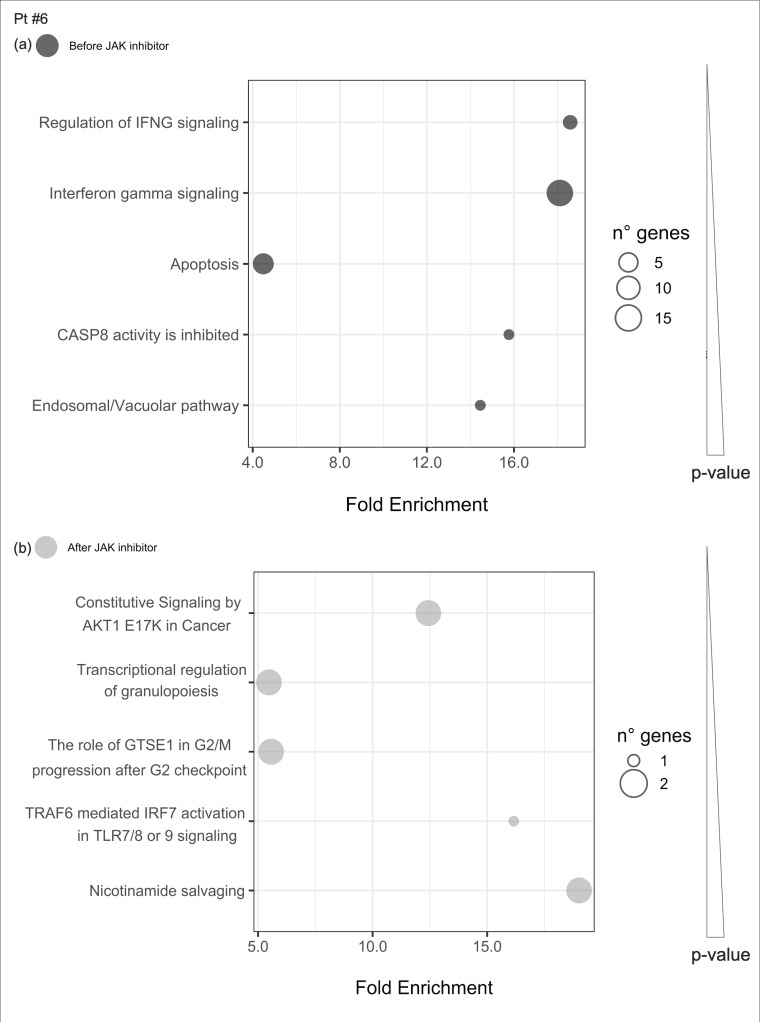
Pathway enrichment analysis was run on differential expressed genes resulting from the comparison between patient #6 before JAKinhibs and the control-group subjects, and between patient #6 after JAKinhibs and the controls. Pathways altered uniquely before (**a**) or uniquely after (**b**) JAKinhibs were chosen. Each dot represents a pathway, and the size is directly proportional to the number of genes. On the *X*-axis the pathway fold enrichment is displayed. All the selected pathways showed a lowest adjusted *p*-value of the given term over all iterations ≤0.01, and the five most enriched pathways were elected, where applicable. Pathways are ordered by increasing *p*-values. Pathways altered before JAKinhibs are colored in dark grey and those altered after JAKinhibs in light grey.

**Figure 14:**
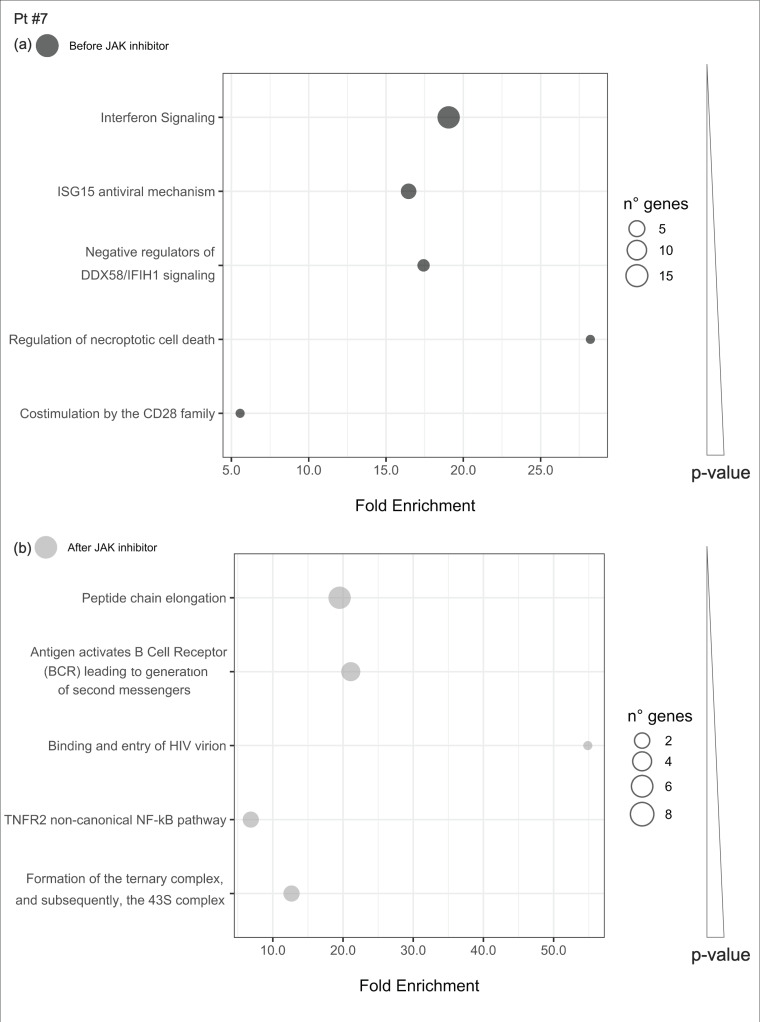
Pathway enrichment analysis was run on differential expressed genes resulting from the comparison between patient #7 before JAKinhibs and the control-group subjects and between patient #7 after JAKinhibs and the controls. Pathways altered uniquely before (**a**) or uniquely after (**b**) JAKinhibs were chosen. Each dot represents a pathway, and the size is directly proportional to the number of genes. On the *X*-axis the pathway fold enrichment is displayed. All the selected pathways showed a lowest adjusted *p*-value of the given term over all iterations ≤0.01, and the five most enriched pathways were elected, where applicable. Pathways are ordered by increasing *p*-values. Pathways altered before JAKinhibs are colored in dark grey and those altered after JAKinhibs in light grey.

Uniquely enriched pathways before or after JAKinhibs were selected in each patient to investigate the modulation of the most representative signaling during the treatment (from Figure 8, Figure 9, Figure 10, Figure 11, Figure 12, Figure 13 and Figure 14).

Altogether the results showed no shared pathways between all the patients after JAKinhibs treatment. However, in four out of seven patients (patients #1, #4, #5, and #7) signaling involved in protein metabolism are found to be commonly enriched. This data was confirmed also with other two gene ontology software (STRING [7], and DAVID [8,9]).

To give an insight also at the gene level, beside pathway analysis, Table 2 displays significantly differentially expressed genes endorsed by more patients only after JAKinhibs treatments.

### 2.5. Concomitant Medications and Reported Adverse Events

The treatment was well-tolerated in all patients and none had to discontinue the medication during the follow-up. However, patient #1 experienced adverse events that led to brief transitory reduction of the drug dosage. After six months of treatment, he developed pulmonary arterial hypertension, with questionable relationship with the treatment, as he improved afterwards by increasing the dose of ruxolitinib together with starting vasodilators [10]. One year later, he developed shingles at the root of the left thigh, which did not require hospitalization and recovered in two weeks with antiviral treatment. Patient #2 developed dyslipidemia that, however, was well managed by adding low dose atorvastatin without changing the dosage of the JAKinhib.

BK virus and JC virus viremia and viruria were measured in all patients at baseline and during follow-up: none showed activation of JC virus replication in blood or urine; urinary BK virus was detectable during the treatment in three out of seven subjects at medium titers (1.8–2.8 × 10^4^ copies/mL), without any sign or renal disease; replication of BK virus in peripheral blood was detected at low titer (1.8 × 10^2^ copies/(mL) in one patient (#3), who is currently being monitored without changes in the JAKinhib dose.

## 3. Discussion

Despite the great efforts made in the last decade to authorize medicines for pediatric use [11], the treatment of inflammatory disorders in children largely relies on off-label use of drugs developed for adults [12]. This is especially true when dealing with severe rare diseases [13]. At the same time, great attention has been given to the opportunity of developing precision medicine approaches driven by the knowledge of mechanisms involved in disease pathogenesis. Subjects with rare monogenic disorders provide simplified models for pathogenesis-targeted therapies [14,15].

In multifactorial rheumatic conditions, genomic and transcriptomic analyses have been proposed for disease stratification and selection of meaningful treatments [16,17]. Unfortunately, pediatric patients with severe rheumatologic conditions are less likely to benefit from these studies, since the drugs available for children are much less than for adults. According to recent data, up to 8% of adverse drug reactions are associated with the off-label administration of medications in Italy [18]. Consequently, it is even more important to get, as much as possible, safety data and biological correlates when proposing off-label treatments to children. 

We describe off-label use of JAKinhibs in a pediatric series of patients affected with severe, refractory rheumatologic conditions, addressing both safety issues and biological changes. 

All patients enrolled in the study were affected with severe inflammatory disorders that had proven refractory to conventional medications, including biologic agents. A positive IFN signature at the baseline was a prerequisite for treatment with JAKinhibs, both as a rationale principle, and as a putative predictor of safety. Even if JAKinhibs exert their action targeting multiple signaling pathways, their property of inhibiting IFN-mediated inflammation is almost unique among antirheumatic drugs [19]. Thus, these drugs may give a significant advantage in subjects refractory to other medications, who have increased IFN-mediated inflammation, as assessed by transcriptomic studies such as the measure of the IFN signature score [20]. However, since IFNs are crucial in the immune response to viruses and mycobacteria, the inhibition of these cytokines may be harmful. Interestingly, the risk of infections seems only slightly increased in subjects with monogenic interferonopathies despite treatment with high doses JAKinhibs, probably because a complete inhibition of the IFN signaling is not achieved, as indicated by the little or partial reduction of the IFN score observed in treated patients [21,22]. Thus, a high baseline IFN score might predict a safer profile of JAKinhibs. 

Indeed, no patient in our series developed serious infections during follow-up: in only one case, herpes zoster reactivation occurred, which was easily managed by reducing the drug dosage and by adding antiviral therapy with acyclovir. High IFN score was suppressed during treatment in four subjects, whilst it remained high in the other three. The reduction of the IFN score was even more notable considering that it occurred also in some patients who concomitantly reduced their dosage of glucocorticoids. However, clinical improvements recorded in the patients didn’t correlate directly with the decrease of IFN score. Indeed, even if measuring of IFN score can help disease stratification, it seems scarcely relevant to assess disease activity during follow-up as in the case of SLE or in some monogenic interferonopathies [21,23]. Moreover, a high IFN score has been described in healthy relatives of patients with SLE, suggesting that it is not sufficient to cause pathology [24].

For this reason, we studied other transcriptomic pathways that were deregulated in patients compared with controls and evaluated how they changed during the treatment. 

Transcriptomic analysis in our series confirmed a primary involvement of IFN-related pathways in patients, which was an expected result given that an increased IFN score was a prerequisite to access off-label use of JAKinhibs at our hospital. However, as a whole, the group of patients continued to cluster differently from healthy controls at baseline, also after the removal of IFN-related pathways from the analysis. At baseline, there were several pathways containing genes differentially expressed in patients compared with controls. For example, DNA repair pathway was upregulated in patients (with increased expression of *BRCA2* and *RMI2*), probably reflecting DNA damage induced by inflammation-derived reactive oxygen and nitrogen species [25,26]. Interesting, mitochondrial DNA damage occurring in chronic inflammatory diseases can contribute to a positive feedback of inflammation, by stimulating the IFN pathway, and JAKinhibs have been proposed as possible anti-inflammatory agents in these settings [27]. Indeed, this pathway was no longer significantly upregulated in patients after the treatment. 

Other altered pathways at baseline include extracellular collagen homeostasis (with several gene downregulated), possibly due to an extracellular matrix catabolic signature of inflammatory macrophages [28]. The involvement of amyloid pathway and of DDX58/IFIH1 pathways is also coherent with the inflammatory status of patients, as the expression of *DDX58* correlates with IFN score in patients with autoimmune rheumatic diseases [29]. The downregulation of genes in the PI3K pathway (*C-Kit* and *PDGFB*) may also reflect an indirect effect of IFN-mediated inflammation [30].

None of these pathways remained significantly involved after therapy in our patients. Indeed, after treatment, the distance between patients and controls was shortened, with a reduced number of differentially involved pathways. These results may suggest that JAKinhibs influenced other pathogenic mechanisms relevant to the selected diseases, in addition to the IFN-related ones. 

Considering the huge heterogeneity between patients in our series, as concerns age and diagnosis, we further analyzed gene expression changes for each patient. We distinguished three types of changes: (i) pathways with altered expression at baseline but not after treatment; (ii) pathways with altered expression only after treatment; (iii) pathways with altered expression persistent across the study. We speculated that pathways in group (i) may mainly represent disease-related mechanisms affected by treatment; pathways in group (ii) might represent drug-related changes, possibly associated with adverse events; pathways in group (iii) may correlate with disease type rather than with its activity.

Among pathways with significant alteration only at baseline, the apoptosis pathways emerged with survival genes hyperexpressed both in the patient with monogenic SLE (#1) and in the patient with sporadic SLE (#6); in patient #2, affected with CANDLE syndrome, it was worth noting the downregulation of PI3K-AKT pathway, which might be related to the severe lipodystrophy of the girl, if we hypothesize that a similar change can occur in adipocytes as well [31]; in patients #4, #5, #6, #7, IFN-related signaling is one of the main pathway significantly altered at baseline.

No pathway was consistently altered in all the patients after treatment, suggesting that there is no common transcriptomic change induced by the treatment in all the seven patients.

Among pathways altered all across the study, IFN signaling was increased in a patient with monogenic SLE and in a patient with sporadic SLE. Consistently, the two patients (#1 and #6) maintained a high IFN score after the treatment. This may not be surprising as it is known that the IFN score does not correlate with disease activity in SLE [23]. Other pathways were altered across the study in other patients, but the differentially expressed genes involved changed after treatment, making their significance in the disease nonobvious. 

We acknowledge that our study has several possible limitations. The case series was quite small and heterogeneous; however, we believe that it is worthwhile to describe experiences concerning rare diseases even in small series. An altered expression of distinct pathways containing IFN-stimulated genes was demonstrated in all patients. In addition, other pathways emerged that could be relevant to the disease pathogenesis and to the response to treatments. This is cross-sectional noninterventional study; thus, patients could change concomitant treatments according to clinical needs. Actually, this was a limitation also in other studies involving patients with rare or complex disease, where the priority is personalization of therapy [21]. Even with these limitations, our study provides valuable data that can be merged with other small series to yield more significant results, in a sort of multicenter virtual trial.

## 4. Materials and Methods

The study is part of the IRCCS Burlo Garofolo project RC #24/2017, approved by the Institutional Review Board and by the Friuli Venezia Giulia Independent Ethical Committee (2018-SPER-079-BURLO, N. 0039851, approved on 12 December 2018). 

### 4.1. Off-Label Use of JAK Inhibitors

We included all the subjects who received JAKinhibs as off-label medication in the Pediatric Department of the IRCCS Burlo Garofolo from January 2015 to May 2020.

Off-label use was evaluated in each case according to a multidisciplinary discussion of the rationale upon the proposal and the available literature. According to Italian law, off-label use of medication can be granted when the following conditions are met: disease refractory to conventional available treatments; published phase II clinical trials showing efficacy and safety in the same condition; safety data available in children in the same or in a different clinical indication; informed consent by the guardian; and assumption of responsibility by the physician who prescribes the medication. 

In particular cases, when dealing with rare disorders, off-label use can be authorized in the absence of phase II studies in the same condition, basing the decision on a principle of clinical and pathological similarity with more common disorders occurring in children and in adults. 

Considering that one added value of JAKinhibs is their capacity to target IFN inflammation, we selected for treatment only subjects with a baseline positive IFN signature in peripheral blood who didn’t achieve an acceptable control of the disease with conventional treatments. Moreover, previous reports showed that JAKinhibs didn’t achieve a complete IFN suppression in subjects with interferonopathies, suggesting that their use could be safer the higher the baseline IFN activation is. 

Safety data of JAKinhibs in children were derived from clinical trials in juvenile idiopathic arthritis and from reports describing experiences in rare monogenic disorders. 

In all cases the prescription is considered off-label “per age” and in all but one patient also “per indication”. 

Off-label treatment with JAKinhibs are monitored according to an internal protocol with baseline and follow-up evaluations, including cardiologic and pneumological assessments, virus serology and PCR in blood and urine and serum lipid profiling. 

For each patient included in the study, we obtained a signed informed consent to receive an off-label medication. Clinical and laboratory data were recorded in a structured database, according to the current European General Data Protection Regulation.

### 4.2. Patients

The study was proposed to all the patients who received off-label prescription of a JAKinhibs for a rheumatologic condition at the Department of Pediatric of the IRCCS Burlo Garofolo. As per law, the patient’s parents/guardian were requested to sign an informed consent for acceptance of the off-label treatment. In addition, they were requested to sign also a separate consent to participate in the research project RC24/17, which aimed at describing how clinical, genetic and transcriptomic data can predict the response to targeted therapies in pediatric immune disorders. 

Data from all the patients were collected in a structured database, reporting clinical and laboratory parameters, type and dosage of the administered JAKinhib, previous and concomitant medication. All serious adverse events were also reported. Erythrocyte sedimentation rate, C reactive protein, immunoglobulin levels, blood cell count and differential were correlated with clinical and transcriptomic changes. Laboratory findings (before and after JAKinhib) were represented by GraphPad Prism 8 software.

### 4.3. Sample Collection, RNA Isolation and cDNA Preparation

Peripheral blood was collected in PAXgene Blood RNA Tubes (PreAnalytiX, Hombrechtikon, Switzerland) and, after two hours’ incubation at room temperature, tubes were frozen at −20 °C until processing. Total RNA was extracted with PAXgene Blood RNA Kit (PreAnalytiX, Hombrechtikon, Switzerland), following the manufacturer’s instructions, and quantified with NanoDrop Spectrophotometer (Thermo Fisher, Waltham, MA, USA). RNA integrity was checked using an Agilent Technologies 2100 Bioanalyzer.

Up to 1 μg of total RNA was retrotranscribed using SensiFAST cDNA Synthesis Kit (Bioline, London, UK).

### 4.4. IFN Signature Analysis

The expression of six IFN stimulated genes was assessed by qPCR using AB 7500 Real Time PCR System (Applied Biosystems, Waltham, MA, USA), TaqMan Gene Expression Master Mix (Applied Biosystems, USA) and UPL Probes (Roche, Basel, Switzerland) for *IFI27*, *IFI44L*, *IFIT1*, *ISG15*, *RSAD2*, and *SIGLEC1*. Using AB 7500 Real Time PCR software, each target quantity was normalized with the expression level of *HPRT1* and *G6PD*, and the relative quantification (RQ) was conducted relating to a “calibrator” sample (mix of ten control-group subjects, five males and five females) using the 2^−∆∆Ct^ method [32]. The median fold change of the six genes was used to calculate the IFN score for each patient.

### 4.5. RNAseq Analysis

Transcriptome sequencing was performed using the TruSeq Stranded mRNA Sample Preparation kit (Illumina, San Diego, CA, USA) and sequenced on a NovaSeq 6000 platform (Illumina, San Diego, CA, USA), generating 2 × 100 bp paired-end reads (30 million reads per sample or 60 million reads per sample) in seven subjects pre- and post-treatment (one sampling for each condition) with JAKinhibs and six young control-group subjects (three males and three females among the ten used for qPCR). RNAseq sample are displayed in Table 3.

RNAseq raw data workflow was conducted as follows: quality control by FastQC (https://www.bioinformatics.babraham.ac.uk/projects/fastqc/), quality filtering by Trim Galore (https://www.bioinformatics.babraham.ac.uk/projects/trim_galore/), read alignment to hg38 using annotation from GENECODE v.34 (https://www.gencodegenes.org/) with STAR [33], reads counting into genes by featureCounts [34].

Data were normalized and analyzed for differentially expressed genes by DESeq2 [35]. Genes that had the sum of the reads ≤10, across all sample, were removed. Differential gene expression analyses were performed comparing data of patients with rheumatologic diseases before and after JAKinhibs with control-group individuals. The same analysis was run correlating each patient with gender-matched control-group subjects both before and after JAKinhibs. Representative genes were selected by fold change greater than twofold increase/decrease and adjusted *p*-value < 0.05, according to the Benjamin–Hochberg method [36].

### 4.6. Cluster Analysis

Cluster analysis was performed using the unsupervised machine learning algorithm K-Means clustering [37] provided by R (http://www.R-project.org/). This analysis algorithm partitions patients into subgroups characterized by similar gene expression patterns, apart from IFN signaling strongly represented in the majority of patients. Before running the k-means algorithm, the optimal number of clusters was defined by the average silhouette method that measures the quality of clusters. The number of random starting partitions (nstart) was set to 40. The most common IFN-related genes were indeed excluded from the examination. IFN-related genes were selected according to literature data [5,6] and to a preliminary gene ontology analysis (Reactome database) of DEGs (between patients and healthy subjects) focusing on IFN signaling pathways. In this case, the expression of 500 most variable protein coding genes across all samples was considered. Clustering results were visualized employing the R functions fviz_cluster (“factoextra package”) that performs the PCA. Data are plotted according to the two and principal components (Dim1 and Dim2) that describe the larger part of the variance between the clusters.

### 4.7. Pathway Enrichment Analysis

To investigate the involvement of potential predominant pathways selected differentially expressed genes were analyzed for pathway enrichment by running the R package pathfindR (*p*-value threshold < 0.05) [38], according to the Reactome database. The output describes:

- The fold enrichment value calculated considering the list of enriched genes in a specific pathway and the number of total input genes.
- The *p*-value that assesses the strength of the association between the genes of interest and the pathway is not random. The smaller the *p*-value, the more the probability that the overrepresentation of certain pathways might underline a real biological effect.

The hierarchical cluster of pathfindR enrichment results was performed employing “cluster_enriched_terms” function and only representative pathways were sorted to eliminate possible biological redundant signaling. 

Pathway enrichment analysis was carried out to identify:

- The common pathways between all the rheumatologic patients both before and after JAKinhibs compared to control-group subjects. The most common IFN-stimulated genes [5,6] were excluded from the analysis.
- The most enhanced shared pathways before and after the JAKinhib in each patient to evaluate signaling that may be independent of this pharmacological treatment.

Pathways were selected considering the lowest adjusted *p*-value of the given term overall iterations ≤0.01 and selected the five most significant (if present), representative of both before and after treatment resulting after running clustering function to eliminate possible biological redundant signaling.

- The enriched pathways either before or after JAK treatments in each patient considering up to five signaling, ranked according to their lowest adjusted *p*-value to examine the possible modulation of JAKinhibs.

Gene ontology analysis was run again on other two software, STRING [7] and DAVID [8,9] to identify possible common pathways in all/some patients related to a possible conserved transcriptional mechanism of JAKinhibs which may not be identified by the previous examination.

### 4.8. Common DEGs and Pathways Altered Only after JAKinhibs

Common DEGs between two or more patients were selected with a stricter cut-off (fold change greater than fourfold increase/decrease) and a gene ontology analysis was run again on other two software, STRING [7] and DAVID [8,9] to identify possible common gene and pathways in all/some patients related to a possible conserved transcriptional mechanism of JAKinihibs which may not be identified by the previous examination.

## 5. Conclusions

The IFN pathway represents an attractive target for treating complex IFN-driven disorders. Nowadays, JAKinhibs are among the most effective medications contrasting IFN inflammation. They seem to be more potent compared with more specific blockers of IFN signaling blockade, such as anifrolumab, a monoclonal antibody blocking the Type I IFN receptor. Despite the encouraging results obtained in recent trials (the secondary end point of the phase III trial provides evidence of the efficacy in SLE), anifrolumab has not achieved the desired modulatory impact, by balancing benefits and adverse effects [39]. 

The correlation of clinical and biological data can serve not only to evaluate what biological changes parallel clinical improvement but can also provide useful data to help patient stratification for therapies in clinical trials or for off-label use of medication in severe and complex cases.

## Figures and Tables

**Table 1:** Characteristics of the patients and medications.

Pt	Diagnosis and Symptoms	Medications			
		Previous Medications	JAKinhib	Concomitant Medications	Adverse Events
#1	Monogenic lupus (DNase2 deficiency) Lipodystrophy, arthritis, lupus pernio, distal ulcers, hepatosplenomegaly, recurrent fever, growth deficiency	Corticosteroids, methotrexate, mycophenolate, hydroxychloroquine, etanercept, anakinra, thalidomide, canakinumab, abatacept	Ruxolitinib (7.5 mg/BID)	Corticosteroids, hydroxychloroquine, mepacrine, ambrisentan, tadalafil, iloprost, furosemide, cotrimoxazole	Worsening of lymphopenia
#2	CANDLE Lipodystrophy, arthritis, lupus pernio, distal ulcers, growth deficiency	Cyclosporine, anakinra, IVIG, cyclophosphamide, azathioprine, infliximab, hydroxychloroquine, abatacept, hyperbaric oxygen	Tofacitinib (7.5 mg/BID)	Atorvastatin, corticosteroids	Transient increase of gamma glutamyltransferase and dyslipidemia
#3	COPA Arthritis, cough	Corticosteroids, mycophenolate, hydroxychloroquine, methotrexate	Baricitinib (2 mg/BID)	Mycophenolate, corticosteroids	None
#4	Polyarticular JIA Arthritis, articular deformity	Corticosteroids, methotrexate, etanercept, tocilizumab	Tofacitinib (5 mg/BID)	Methotrexate	None
#5	Weber-Christian Panniculitis Recurrent panniculitis, recurrent fever, arthralgia, fatigue	Corticosteroids, mycophenolate, cyclosporine, anakinra	Baricitinib (2 mg/TID)	Cyclosporine, cotrimoxazole	None
#6	SLE Headache, fatigue, social isolation, fever, malar rash	Corticosteroids, mycophenolate, hydroxychloroquine	Baricitinib (4 mg/QD)	Mycophenolate, Hydroxychloroquine	None
#7	Juvenile systemic sclerosis Raynaud, distal ulcers, stiffness, arthritis	Mycophenolate, corticosteroids, rituximab *	Tofacitinib (5 mg/BID)	Mycophenolate, corticosteroids	None

Pt, patient; CANDLE, chronic atypical neutrophilic dermatosis with lipodystrophy and elevated temperature; IVIG, intravenous immunoglobulins; BID, bidaily; JIA, juvenile idiopathic arthritis; TID, tridaily, SLE, systemic lupus erythematosus; QD, once a day. * The baseline sample considered in the later RNAseq analysis was collected before starting any pharmacological treatment (medication-naive), in contrast to the other patients.

**Table 2:** Common differentially expressed genes (DEGs) altered only after JAKinhibs.

Gene	Patient #1	Patient #2	Patient #3	Patient #4	Patient #5	Patient #6	Patient #7
*BCL2A1*			Up-reg	Up-reg			
*CAMP*		Up-reg			Up-reg		
*CTNNAL1*	Up-reg	Up-reg					
*DEFA3*		Up-reg					Up-reg
*EEF1B2*				Up-reg	Up-reg		
*FBXO7*	Up-reg	Up-reg					
*FCRL1*			Up-reg			Up-reg	
*GNG11*				Up-reg	Up-reg		
*HAGH*	Up-reg	Up-reg					
*HP*		Up-reg	Up-reg				
*IFITM3*		Down-reg	Up-reg				
*IGF2BP2*	Up-reg	Up-reg					
*LGALS3*	Up-reg	Up-reg					
*LTF*		Up-reg				Down-reg	
*MBNL3*	Up-reg	Up-reg					
*MXI1*	Up-reg	Up-reg					
*PAX5*			Up-reg			Up-reg	
*PDCD10*			Up-reg	Up-reg			
*PDK4*		Up-reg		Up-reg			
*PLEKHG1*			Up-reg			Up-reg	
*RPL41*				Up-reg			Up-reg
*RPS21*				Up-reg	Up-reg		
*RPS29*				Up-reg	Up-reg		
*RSL24D1*			Up-reg	Up-reg	Up-reg		
*SIAH2*	Up-reg	Up-reg					
*SLC14A1*	Up-reg	Up-reg					
*TMA7*				Up-reg	Up-reg		
*VWCE*		Up-reg	Down-reg				
*ZNF117*	Up-reg		Up-reg				

Up-reg, upregulated gene. Down-reg, downregulated gene. Genes are reported in italics. Background colors display genes expressed in different patients: patient #1 in green, patient #2 in pink, patient #3 in light blue, patient #4 in orange, patient #5 in blue, patient #6 in red, and patient #7 in grey.

**Table 3:** RNAseq samples.

Datasets	Subjects		RNAseq Details		
Sample/*n*	Female (*n* = 9)	Male (*n* = 4)	Whole Blood Collection/RNA Extraction	RNAseq Library Preparation/Platform	Read Length/Coverage
Rheumatologic patient sample before JAKinhibs/ *n* = 7	6	1	PAXgene blood RNA tube/PAXgene Blood RNA Kit	Illumina TruSeq stranded mRNA library protocol/Novaseq	Paired-end 100 bp reads/ 30 M or 60 M reads
Rheumatologic patient sample after JAKinhibs/ *n* = 7					
Healthy subjects/ *n* = 6	3	3			

## Supplementary Materials

Supplementary Materials can be found at https://www.mdpi.com/1422-0067/21/20/7767/s1. Table S1. List of genes resulted from single patient pathway analysis.

## Abbreviations

BCL2A1	BCL2 Related Protein A1
BRCA2	Breast cancer gene 2
CAMP	Cathelicidin Antimicrobial Peptide
CANDLE	Chronic atypical neutrophilic dermatosis with lipodystrophy and elevated temperature
COPA	COPI coat complex subunit alpha
CRP	C-reactive protein
CSF	Cerebrospinal fluid
CTNNAL1	Catenin Alpha Like 1
DEFA3	Defensin Alpha 3
DEG	Differential expressed genes
EEF1B2	Eukaryotic Translation Elongation Factor 1 Beta 2
ESR	Erythrocyte sedimentation rate
FBXO7	F-Box Protein 7
FCRL1	Fc Receptor Like 1
FUP	Follow-up
G6PD	Glucose-6-phosphate dehydrogenase
GNG11	G Protein Subunit Gamma 11
HAGH	Hydroxyacylglutathione Hydrolase
HB	Hemoglobin
HP	Haptoglobin
HPRT1	Hypoxanthine phosphoribosyl transferase 1
JIA	Juvenile idiopathic arthritis
JAK	Janus kinase
IFI27	Interferon alpha inducible protein 27
IFI44L	Interferon inducible protein 44 like
IFIH1	Interferon induced with helicase C domain 1
IFIT1	Interferon induced protein with tetratricopeptide repeats 1
IFITM3	Interferon Induced Transmembrane Protein 3
IFN	Interferon
IFNGR1	Interferon gamma receptor 1
IGF2BP2	Insulin Like Growth Factor 2 MRNA Binding Protein 2
IL1R	Interleukin 1 receptor
IL1RAP	Interleukin 1 receptor accessory protein
IS	Interferon score
ISG15	Interferon stimulated gene 15
LGALS3	Galectin 3
LTF	Lactotransferrin
MAPK	Mitogen-activated protein kinase
MBNL3	Muscleblind Like Splicing Regulator 3
MRI	Magnetic resonance imaging
MXI1	MAX Interactor 1, Dimerization Protein
ND	Not Done
PAX5	Paired Box 5
PDCD10	Programmed Cell Death 10
PDGFB	Platelet derived growth factor subunit B
PDK4	Pyruvate Dehydrogenase Kinase 4
PI3K	Phosphoinositide 3-kinase
PLEKHG1	Pleckstrin Homology And RhoGEF Domain Containing G1
RIG-1	Retinoic acid-inducible gene I
RMI2	RecQ mediated genome instability 2
RPL41	Ribosomal Protein L41
RPS21	Ribosomal Protein S21
RPS29	Ribosomal Protein S29
RSAD2	Radical s-adenosyl methionine domain containing 2
RSL24D1	Ribosomal L24 Domain Containing 1
SIAH2	Siah E3 Ubiquitin Protein Ligase 2
SIGLEC1	Sialic acid binding Ig like lectin 1
SLC14A1	Solute Carrier Family 14 Member 1 (Kidd Blood Group)
SLE	Systemic lupus erythematosus
SSA	Anti-Sjögren’s syndrome type A
STAT1	Signal transducer and activator of transcription 1
TMA7	Translation Machinery Associated 7 Homolog
TNFa	Tumor necrosis factor alpha
UPL	Universal probe library
VWCE	Von Willebrand Factor C And EGF Domains
WBC	White blood cells
ZNF117	Zinc Finger Protein 117

## Appendix A

**Table A1:** Patient #1 laboratory findings.

Pt#1	ESR mm/h	IS	CRP mg/L	WBC/μL	HB g/dL	PLT/μL	IgG mg/dL	IgA mg/dL
Before JAKinhibs	9	83	9.9	3350	12.7	107,000	855	124
After JAKinhibs	13	62	0.3	2610	12.2	146,000	459	69
Last FUP	12	22.4	5.5	1680	14.0	130,000	702	61

**Table A2:** Patient #2 laboratory findings.

Pt#2	ESR mm/h	IS	CRP mg/L	WBC/μL	HB g/dL	PLT/μL	IgG mg/dL	IgA mg/dL
Before JAKinhibs	27	29.1	1.7	5700	11.1	195,000	1645	399
After JAKinhibs	46	1.0	0.2	5110	12.4	337,000	1538	368
Last FUP	22	3.0	0.2	5400	12.9	252,000	1752	314

**Table A3:** Patient #3 laboratory findings.

Pt#3	ESR mm/h	IS	CRP mg/L	WBC/μL	HB g/dL	PLT/μL	IgG mg/dL	IgA mg/dL
Before JAKinhibs	68	33.4	5.2	5320	12.5	357,000	1860	207
After JAKinhibs	60	32.0	2.4	10,220	11.4	318,000	1812	169
Last FUP	20	ND	12.0	ND	ND	ND	ND	ND

**Table A4:** Patient #4 laboratory findings.

Pt#4	ESR mm/h	IS	CRP mg/L	WBC/μL	HB g/dL	PLT/μL	IgG mg/dL	IgA mg/dL
Before JAKinhibs	120	5.5	7.9	8050	11.3	499,000	1480	187
After JAKinhibs	65	2.0	3.7	7390	11.1	361,000	1613	107
Last FUP	38	1.7	1.0	6450	11.8	342,000	1527	91

**Table A5:** Patient #5 laboratory findings.

Pt#5	ESR mm/h	IS	CRP mg/L	WBC/μL	HB g/dL	PLT/μL	IgG mg/dL	IgA mg/dL
Before JAKinhibs	56	3.4	46.6	11,030	12.3	216,000	1870	162
After JAKinhibs	81	2.0	16.0	7650	10.3	362,000	1323	185
Last FUP	69	4.0	11.6	5750	11.3	507,000	1334	172

**Table A6:** Patient #6 laboratory findings.

Pt#6	ESR mm/h	IS	CRP mg/L	WBC/μL	HB g/dL	PLT/μL	IgG mg/dL	IgA mg/dL
Before JAKinhibs	33	38.8	1.4	11,030	14.3	424,000	948	197
After JAKinhibs	11	47.0	0.3	7650	12.2	362,000	953	147
Last FUP	10	44.2	0.3	4270	13.0	359,000	1061	176

**Table A7:** Patient #7 laboratory findings.

Pt#7	ESR mm/h	IS	CRP mg/L	WBC/μL	HB g/dL	PLT/μL	IgG mg/dL	IgA mg/dL
Before JAKinhibs	39	17.7	3.9	6150	12.2	272,000	863	177
After JAKinhibs	12	1.0	0.7	7550	13.0	215,000	901	138
Last FUP	8	1.0	0.3	9100	14.7	174,000	910	125

Pt, patient; Last FUP, last follow-up; ESR, erythrocyte sedimentation rate; IS, IFN score, CRP, C-reactive protein; WBC, white blood cells; HB, hemoglobin; PLT, platelet; IgG, IgA, Immunoglobulins; ND = not done.

## Appendix B

After JAKinhibs transcriptomic profile changes: case series and controls.
