# Supplementary material for: Biological and Clinical Changes in a Pediatric Series Treated with Off-Label JAK Inhibitors
Source: Int J Mol Sci. 2020 Oct 20;21(20):7767. doi: 10.3390/ijms21207767 (PMC7590237; doi:10.3390/ijms21207767)
Supplement: Supplementary file 1 [file ijms-21-07767-s001.pdf]

Table S1. List of genes resulted from single patient pathway analysis.

Gene	pt#1	pt#2	pt#3	pt#6	pt#7
ADAR2					
AGO4					
AKT1					
ARRB2					
B4GALT1					
BIRC2					
BIRC3					
BST2					
CEBPA					
CEBPB					
CREBBP					
CSF3R					
DDX58					
E2F3					
EIF2AK2					
ERF					
ETS2					
FCGR1B					
FURIN					
GBP1					
GBP2					
GBP3					
GBP5					
GBP6					
GZMB					
H2AC6					
H2BC21					
H2BC4					
H3-3A					
H3F3A					
HDAC5					
HERC5					
HLA-G					
IFI27					
IFI35					
IFI6					
IFIT1					
IFIT2					
IFIT3					
IFITM1					
IFITM2					
IFITM3					
IFNGR1					
IL6R					
IL6ST					
IRF7					
ISG15					
ISG20					
JAK2					
LEF1					
MAML3					
MAPK1					
MAPK3					
MDM2					
MT2A					

MX1					
MX2					
MYC					
NCSTN					
NEURL1					
NOTCH1					
NOTCH2					
NUMB					
OAS1					
OAS2					
OAS3					
OASL					
PLXND1					
PML					
PSEN1					
PSMC6					
RARA					
RBPJ					
RBX1					
RPL21					
RPL32					
RPL37					
RPL41					
RPL7					
RPS23					
RPS25					
RPS27A					
RSAD2					
RXRA					
SEL1L					
SOCS1					
SOCS3					
SP1					
SP100					
SPI1					
ST3GAL4					
STAT1					
STAT2					
STAT2					
STAT3					
TAL1					
TBL1X					
TFDP					
TFDP1					
TLE3					
TNFAIP3					
TNFRSF13C					
TNFRSF18					
TNFRSF1A					
TNFRSF4					
TNFRSF9					
TNFSF13B					
TNFSF14					
TRIM10					
TRIM22					
TRIM25					
TRIM38					
TRIM5					
TYK2					

UBA52					
UBB					
UBE2L6					
USP18					
WWP2					
XAF1					

Genes shared by two or more subjects are highlighted. Background colors display genes expressed in different patients: patient #1 in green, patient #2 in pink, patient #3 in light blue, patient #6 in red, and patient #7 in grey.
